# Nanocomposite coatings for the prevention of surface contamination by coronavirus

**DOI:** 10.1371/journal.pone.0272307

**Published:** 2022-08-02

**Authors:** Esti Toledo, Sharon Dim, Avishay Edri, Yariv Greenshpan, Aner Ottolenghi, Nadav Eisner, Sivan Tzadka, Ashish Pandey, Haggai Ben Nun, Guillaume Le Saux, Angel Porgador, Mark Schvartzman

**Affiliations:** 1 Department of Materials Engineering, Ben-Gurion University of the Negev, Beer-Sheva, Israel; 2 Ilse Katz Institute for Nanoscale Science & Technology, Ben-Gurion University of the Negev, Beer-Sheva, Israel; 3 The Shraga Segal Department of Microbiology, Immunology, and Genetics, Faculty of Health Science, Ben-Gurion University of the Negev, Beer-Sheva, Israel; University of Southern Denmark, DENMARK

## Abstract

The current Covid-19 pandemic has a profound impact on all aspects of our lives. Aside from contagion by aerosols, the presence of the SARS-CoV-2 is ubiquitous on surfaces that millions of people handle daily. Therefore, controlling this pandemic involves the reduction of potential infections via contaminated surfaces. We developed antiviral surfaces by preparing suspensions of copper and cupric oxide nanoparticles in two different polymer matrices, poly(methyl methacrylate) and polyepoxide. For total copper contents as low as 5%, the composite material showed remarkable antiviral properties against the HCoV‐OC43 human coronavirus and against a model lentivirus and proved well-resistant to accelerated aging conditions. Importantly, we showed that the Cu/CuO mixture showed optimal performances. This product can be implemented to produce a simple and inexpensive coating with long-term antiviral properties and will open the way to developing surface coatings against a broad spectrum of pathogens including SARS-CoV-2.

## Introduction

The global coronavirus pandemic (COVID-19) has started at the beginning of 2020, and its ending is still uncertain. Despite recent progress in the vaccination against the SARS-CoV-2, the number of infected people and fatal cases is growing, in particular, due to several new variants of the virus [1], whose infectivity is higher than the original virus that spread from China [2]. At the same time, it is broadly agreed that the SARS-CoV-2 is mainly transmitted through infected saliva and respiratory secretions, or through airborne respiratory droplets; the surface-induced transmission is another, less studied factor in the spread of the virus [3, 4]. Recent work has shown that, although low, the risk of infection from contact with a fomite existed, which suggests that fomites could play a role albeit minimal in SARS-CoV-2 community transmission [5]. Also, the indirect transmission of the SARS-CoV-2 *via* nasal-oral route has been reported [6]. Thus, preventing contact-induced transmission of the SARS-CoV-2 is necessary to curtail the current global pandemic [7].

One long-known way to eliminate the contamination of surfaces by viruses and bacteria, and thus a possible contact-induced transmission of these pathogens, is by using metallic surfaces [8]. Metals, such as zinc, silver, titanium oxide and copper, are poisonous to viruses and bacteria, even in small amounts, and are used as antiviral and antibacterial agents in medical applications^7-20^. In particular, copper was recognized in 2008 by the United States Environmental Protection Agency (EPA) as the first metallic antimicrobial agent [9]. In addition to using copper surfaces, which is not always practical, copper can be adsorbed on the surface of cotton fibers, latex, and other polymeric materials [10] in the form of nanoparticles (NPs) [11–16]. The bactericidal effect of these nanoparticles has been attributed to their small size and high surface-to-volume ratio, which allows them to interact closely with microbial membranes and is not solely due to the release of metal ions in solutions [17–19]. Copper nanoparticles embedded into polymers have been shown to eliminate infections by many types of bacteria such as *Staphylococcus aureus*, *Pseudomonas aeruginosa* [20], *E-coli* [21, 22], and several viruses such as the *Novovirus* that causes gastroenteritis [23], *Influenza A* [23], as well as the *Monkeypox virus* and *Vaccinia virus* [24]. However, all the systems mentioned above base their antimicrobial activity on the release of copper ions from the substrate into the solution, which, like other drug-eluting systems, has significant drawbacks: (i) ion release can lead to a short-term lifetime of the coatings, (ii) it is complicated to control the release of the ions accurately [25], and (iii) copper oxide can be cytotoxic [26]. These have implications for real-life applications that require coatings with constant antimicrobial properties. More importantly, as of today, the is no report of antiviral surfaces which specifically target the SARS-CoV-2. In light of the current situation, it is urgent to find solutions to mitigate the COVID-19 pandemic.

This work reveals the potential of Cu nanoparticles embedded into a polymer matrix to effectively minimize the presence of surface-bound coronavirus in the long term. We studied the HCoV-OC43 human coronavirus whose clinical presentation resembles that of SARS‐COV‐2 [27]. This strongly suggests that our findings on the antiviral efficacy of our coatings are also applicable to SARS-CoV-2. The composite makes it suitable to coat a wide variety of surfaces due to the polymers’ adhesive nature and the wide range of choices available. Therefore, Cu nanoparticle-polymer composites can be optimized for the rapid and easy coating of daily used surfaces such as door handles and elevator knobs, that are potential transmission sources.

In this paper, we explore the prevention of the surface contamination of HCoV-OC43 and a model lentivirus by nanocomposite coatings based on Cu and CuO nanoparticles embedded into two types of polymers. Here, the polymers serve as a stabilizing matrix for the nanoparticles and also facilitate the production of coatings onto surfaces. We demonstrate that the composition of such Cu/polymer composite coatings could be optimized to produce an almost complete inactivation of the virus combined with high transparency and long-term durability. We particularly examined the combination of Cu and CuO nanoparticles with poly(methyl methacrylate) (PMMA) and epoxy-based polymers at different concentrations. We found that the two polymers produce strikingly different antiviral efficiencies. Whereas PMMA based nanocomposites reduced the viral infection to a few percents, the epoxy-based composite was ineffective for viral inactivation, even at high nanoparticle concentrations. We found that the antiviral efficiency of PMMA is associated with a low affinity for the nanoparticles, which causes a significant accumulation of the nanoparticles at the polymer surface, where they can directly contact the virus and kill it. We found that after the accelerated aging tests, the PMMA-Cu nanoparticle composite coatings are able to retain their antiviral activity for long periods. Furthermore, we also found that the optimized formulation of Cu-PMMA nanocomposite has generic antiviral properties by demonstrating its efficiency in the inactivation of another virus of the *Lentivirus* family. This work opens a pathway to novel hybrid coatings for the effective and large-scale prevention of the surface spreading of viruses, including SARS-CoV-2.

## Results and discussion

**Fig 1:**
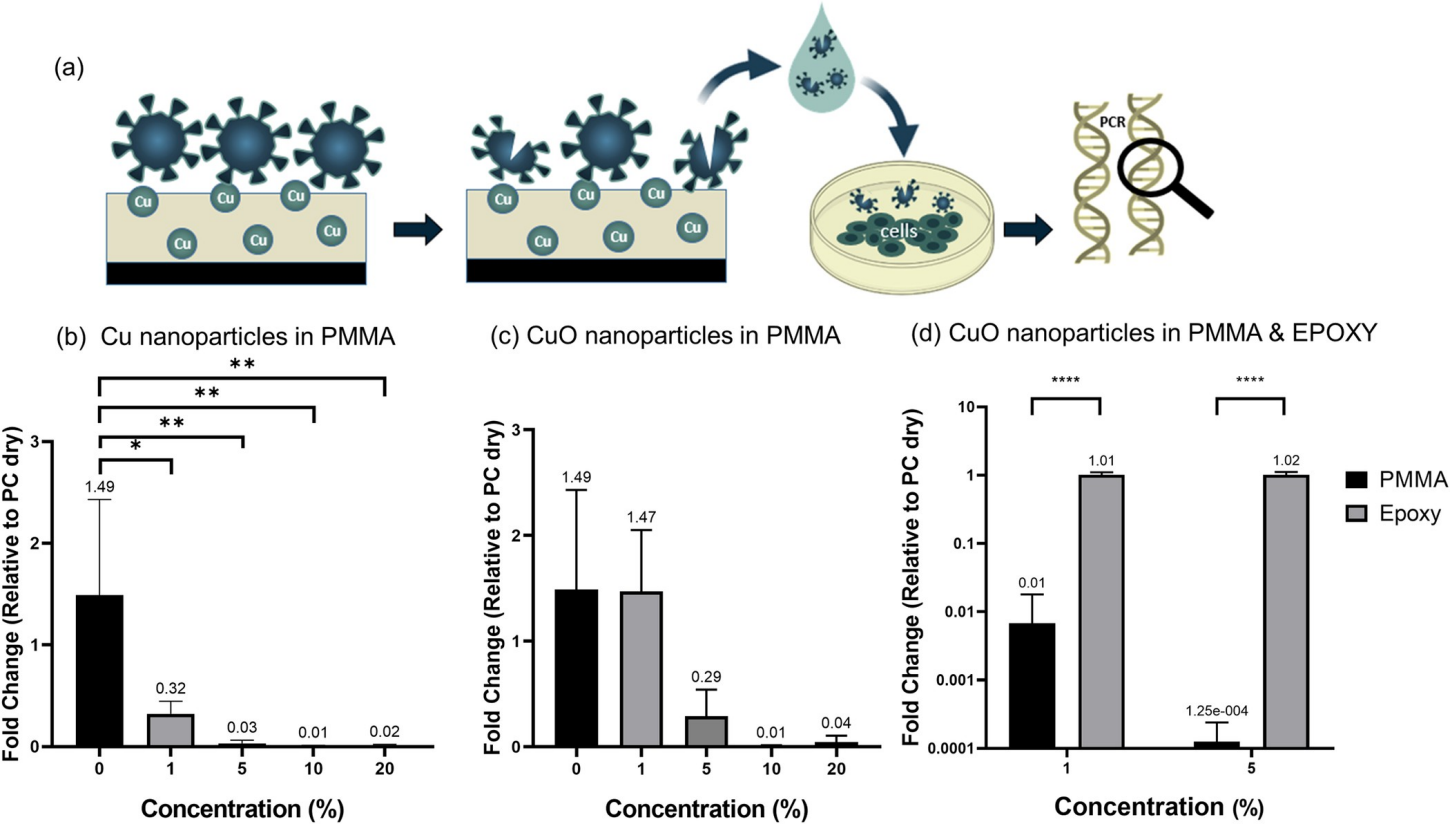
(a) Schematic description of the experimental protocol for the assessment of the antiviral efficiency of the coating (b) antiviral effect of copper NPs suspended in PMMA, (c) effect of CuO NPs suspended in PMMA on viral content (d) antiviral effect of the polymer matrix. Experiments were performed in triplicate. Analysis of variance with a Tukey *post-hoc* test was performed for statistical analyses. Differences between datasets were considered significant for p<0.05.

We assessed the antiviral efficiency of Cu and CuO nanoparticles by embedding them into two commonly used polymers: epoxy (Novolac) and poly(methyl methacrylate) (PMMA). We then added the nanoparticles to the polymer solution in different amounts to obtain nanoparticle concentrations in the range of 1–20% (w/w). The polymers were coated onto inert surfaces to produce films on which we then tested virus inactivation by immobilizing HCoV-OC43 from a solution of viral stock (Fig 1A). To quantify the degree of viral activity of the immobilized Coronavirus, we then incubated the surfaces in cell culture medium and added this medium to cultured HCT-8 cells. After two days of culture, the cells were collected, their RNA was extracted, and the amount of virus was detected using qRT-PCR (details on the experimental procedures are shown in the materials and methods section).

The virus level for the cells cultured in the media collected from the surfaces coated with Cu and CuO nanoparticles in PMMA is shown in Fig 1B and 1C. The level was normalized to the cells cultured in the medium collected from a control sample. First, it can be seen that both types of nanoparticles produce a clear antiviral effect. For pristine PMMA, the amount of virus was slightly higher than that of the control sample. Yet, adding quantities as low as 1% of Cu nanoparticles was sufficient to reduce the virus levels by 79%.

Further increase in Cu concentration produced a dramatic decrease in the infection, with virus levels reduced by 98% for the maximal tested concentration of nanoparticles of 20% (Fig 1B). The trends in the antiviral effect of CuO nanoparticles are similar to that of Cu nanoparticles, albeit to a lesser extent since reductions in viral load higher than 95% were only observed for relatively high concentrations of 10% and 20%. Furthermore, the antiviral effect of CuO is already less pronounced for 5% and completely vanishes for 1%. Finally, the effect of the polymer matrix on the antiviral properties of the coating is shown in Fig 1D. The virus levels observed for the PMMA-based polymer reveal that PMMA was drastically more efficient than the epoxy-based matrix in reducing virus levels. In particular, we found that regardless of copper oxide concentration, the levels of infected cells on PMMA were significantly lower than on epoxy, with a notable three orders of magnitude difference in the case of matrices loaded with 5% copper (ii) oxide.

**Fig 2:**
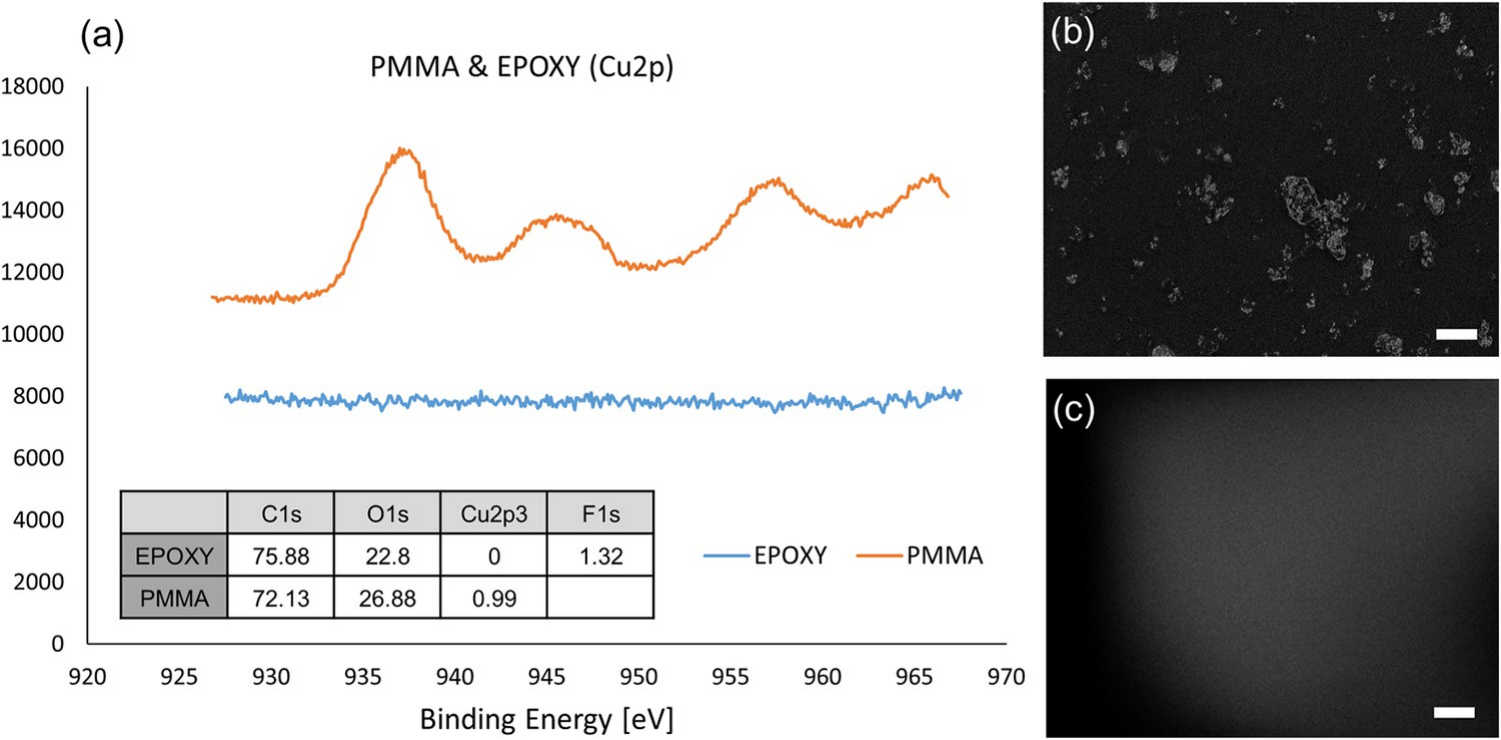
Characterization of samples. (a) XPS results of polymers. (b) SEM images of PMMA solution with 20% CuO NPs (c) SEM images of epoxy solution with 20% CuO NPs. Scale bar: 10 μm.

To find why different polymer matrices produce such strikingly different antiviral effects when mixed with nanoparticles, we characterized the surfaces of the composite films by X-ray photoelectron spectroscopy (XPS). In particular, we aimed at finding whether there was a difference in the amount of the nanoparticles present on the surface of different polymer films. Fig 2 demonstrates the counts/s versus binding energy obtained for PMMA and epoxy mixed with CuO nanoparticles (20%). It follows from the XPS analysis that while there is a substantial amount of CuO present on the surface of PMMA, whose binding energy peaks at 933 eV [28], it is entirely absent on the surface of the epoxy polymer. These results are further confirmed by SEM analyses of both surfaces. CuO nanoparticles are visible on the surface of PMMA while they form agglomerates sized in the range of 1 to 10 microns. However, the agglomeration of the nanoparticles does not hamper their antiviral activity, as seen from the infection assays. Contrary to PMMA, epoxy films showed a surface devoid of nanoparticles. The absence of visible nanoparticles on the epoxy surface mirrors the result obtained by XPS.

The striking difference between the two polymers in terms of the nanoparticle content on their surfaces can be explained through their affinity for Cu and its oxide. It is well known that epoxy polymers bind well to various materials [29] and Cu in particular [30]. Therefore, Cu and CuO nanoparticles would likely produce a homogeneous and strongly bound dispersion within the epoxy matrix, with minimal presence of the nanoparticles to the surfaces. In contrast, PMMA has a relatively poor affinity for Cu and Cu-based compounds [31]. In addition, when sintering PMMA beads with copper NPs, the copper was segregated at the grain boundaries [32]. Therefore, the poor affinity of PMMA for copper can drive the copper to be segregated at boundaries which, in the present case, is the surface of our material. This would then explain the relatively high amount of Cu found on the surface of the PMMA film.[32] Indeed, almost 2% of the surface of a film of a 20% CuO—PMMA mix is covered by the nanoparticles.

Based on this data, we conclude that the observed difference in the antiviral activity between the two polymers stems from the difference in the nanoparticle content at their surface. It should be noted that two mechanisms have been described to explain the antiviral and antibacterial activity of metallic nanoparticles embedded within a polymer matrix. The first mechanism is based on the release of Cu ions and their migration to the surface, where they can interact with adsorbed viruses and bacteria [20]. The second proposed mechanism is based on direct contact between Cu species on the surface and adsorbed viruses [9]. To identify which of these possible mechanisms takes place in our case, we measured the number of Cu ions released from the polymer films. For this purpose, we incubated the surfaces in water for 24 hrs., and then analyzed the water using Inductive Coupled Plasma (ICP) Spectroscopy. To verify that all the possible Cu species in the analytes were ionized, nitric acid was added before the analysis. No Cu^2+^ ions were detected in the supernatants for all the tested surfaces. This finding strongly suggests that in our case, the antiviral effect of the nanoparticles was most probably due to the direct contact with the virus, or “contact killing” [9]. The contact killing mechanism also explains why on epoxy-based surfaces, on which no CuO nanoparticles were present, no antiviral effect was observed.

**Fig 3:**
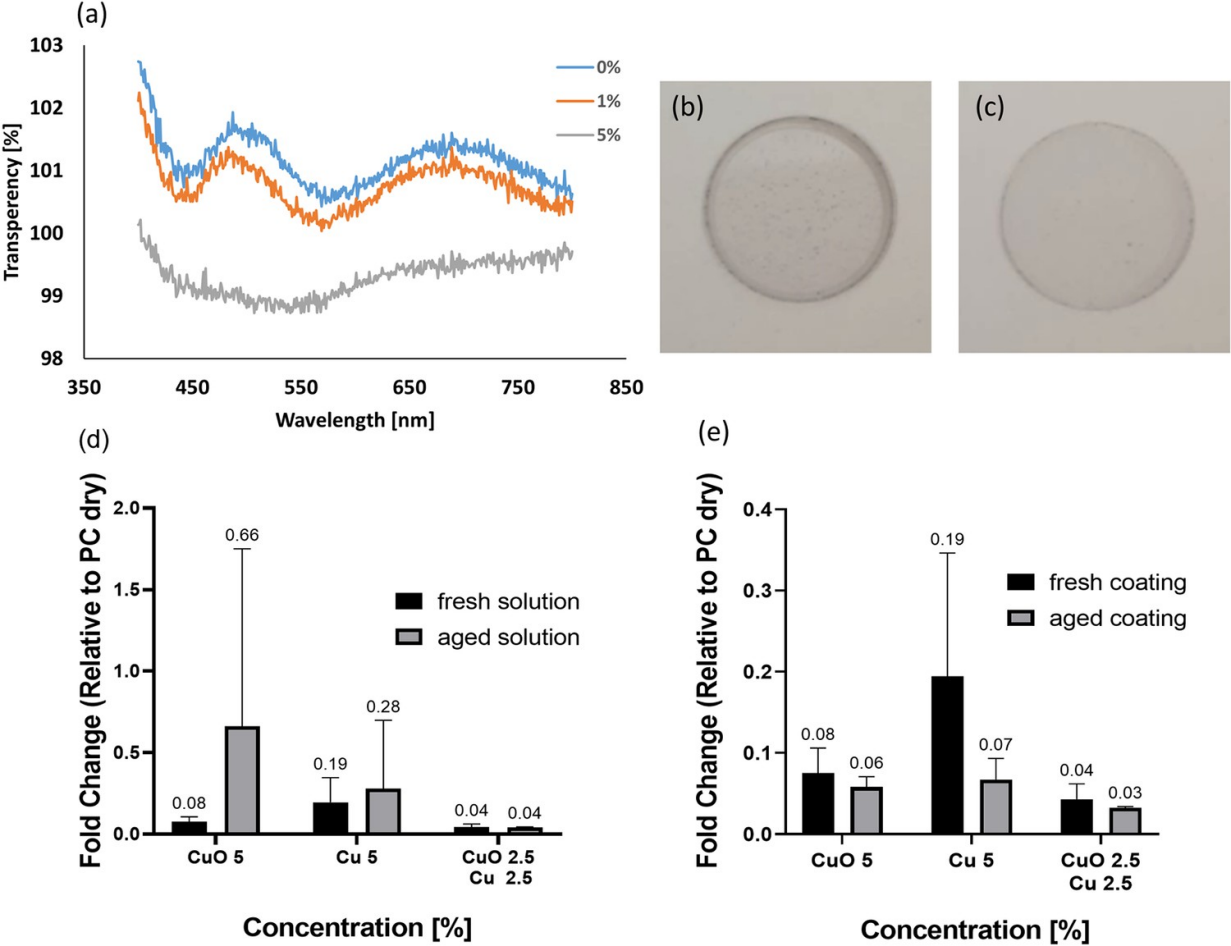
(a) Transparency test of samples of CuO with different concentrations. (b) optical image of PMMA with 10% CuO (c) optical image of PMMA with 5% CuO. (d-e) antiviral effect of freshly prepared or aged composite coatings (d) comparison between the fresh solution and aged solutions (e) comparison between fresh coatings and aged coatings.

The observed antiviral behavior of polymer composites with Cu-based nanoparticles can be applied to engineer surface coatings that can prevent the contamination of surfaces with the SARS-CoV-2 and, therefore, its possible surface-induced transmission. Besides the immediate antiviral activity, as studied in laboratory conditions, such coatings should possess several characteristics for practical applications such as optical transparency. In addition, considering that a commercial product can be stored for long periods before being applied on a surface, the solution of nanoparticles mixed with the polymer should have the longest shelf-life possible. Finally, to ensure a practical lifetime of usage, the coating prepared from this solution should also sustain its antiviral efficiency as long as possible. To address the first requirement, we measured the optical transparency of the films (Fig 3). For this, we chose a CuO NPs concentration of 5%, whose efficiency was demonstrated (Fig 1). We found that the transparency of coated film containing 5% of CuO nanoparticles was 98%.

After we established that 5% is the optimal concentration of CuO-based nanoparticles for both optical transparency and antiviral efficiency, we explored whether and how the aging of (i) a polymer nanoparticle solution and (ii) a prepared film influences their antiviral performance, using two accelerated aging experiments. In the first experiment, a PMMA based solution of Cu nanoparticles, CuO nanoparticles, and 1:1 mixture, at a total concentration of 5% in each case, were aged for 7 days using alternating 12 hr. cycles of heating to 50°C and cooling to room temperature. During the aging experiment, the vessels containing the suspensions were sealed to prevent evaporation of the solvent. The aged suspensions were then used to prepare the antiviral coatings, which were tested as described above. Fig 3D compares the antiviral efficiencies of the films prepared from the aged suspensions to those prepared from the fresh suspensions. Overall, the composite films prepared from the aged suspensions were less efficient than those prepared from fresh suspensions. However, the difference in the degree of elimination was not statistically significant and depended on the film composition. For films with CuO nanoparticles, the solution aging resulted in less than a 1-fold increase in the amount of virus. Conversely, for films with Cu nanoparticles, the solution aging had a very negligible effect on the amount of the virus. Surprisingly, the mix of the two types of nanoparticles, which was tested for the first time in this experiment, yielded a substantially lower virus amount than for individual NP types and showed impressive retention of its antiviral efficiency after solution aging.

In the second experiment, films containing 5% of nanoparticles made of Cu, CuO, or their 1:1 mix, were prepared and aged using the same procedure as described above. Fig 3E presents the antiviral activities of the aged films *versus* that of fresh films. In all cases, the aging did not hamper the antiviral efficiency of the films and even slightly improved it. Again, the mix of Cu and CuO nanoparticles produced the best results, which also improved after aging. The reason for the complementary antiviral effect produced by Cu and CuO nanoparticles is the subject of ongoing research in our team.

The observed ability of Cu and CuO nanoparticles, which were embedded into the polymer matrix, to inactivate SARS-CoV-2 share similar features to that of plain surfaces of Cu or Cu alloys. Remarkably, these surfaces showed effective inactivation of several viruses, most probably through an analogous mechanism [9]. Therefore, it is reasonable to assume that the antiviral activity of the Cu/CuO–polymer matrix could also be general rather than specific to HCoV-OC43. To test whether these nanocomposites’ antiviral efficiency are ubiquitous, we applied the virus elimination assays to a Green fluorescence protein (GFP)-labelled retrovirus variant, henceforth referred to as GFP-lentivirus. The experimental procedure was similar to that used for the HCoV-OC43. Briefly, a diluted stock of retrovirus was seeded on the surfaces covered with the nanocomposite films and on control surfaces. The surfaces were then incubated with a culture medium, and the medium was added to the culture of T cells from the Jurkat cell line. After 48 hrs. of incubation, the cells were harvested and checked by FACS for GFP expression.

**Fig 4:**
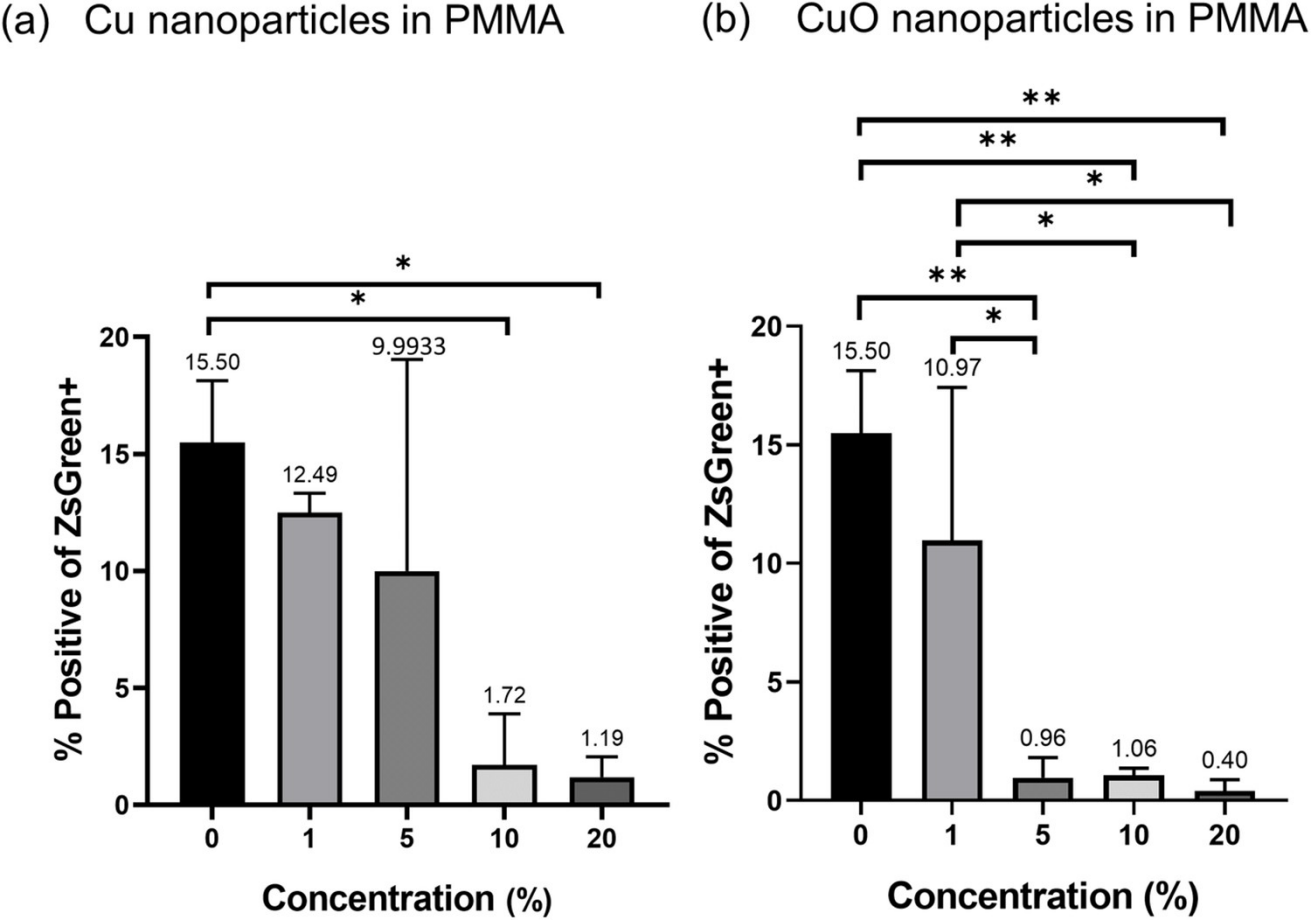
Level of positive of ZsGreen on samples with PMMA solutions (a) samples with different concentrations of copper nanoparticles (b) samples with different concentrations of copper oxide nanoparticles.

Fig 4 shows the normalized amount of GFP content of cells incubated in the supernatant medium collected from polymer films containing different Cu and CuO nanoparticles concentrations. The GFP content was normalized to control surfaces. The observed trends were similar for both nanoparticle types, decreasing the percentage of infected cells with increasing nanoparticle concentration. However, strikingly different efficiencies were observed for a nanoparticle concentration of 5%. While the efficiency of the coating loaded with 5% Cu nanoparticles was not significantly different from the NP free coating, the coating containing 5% CuO showed a drastic decrease in the number of infected cells down to the same level of coatings containing 10 and 20% CuO. Based on these results, it can be concluded that for CuO nanoparticles, the concentration of 5% is a good compromise between transparency and efficiency for which the amount of nanoparticles on the polymer surface is sufficient to inactivate the virus species they contact.

In this work, we demonstrated the effective inactivation of two types of viruses, among which the HCoV-OC43 using polymer composites with Cu and CuO nanoparticles, by a mechanism that most likely involves contact killing. The inactivation of viruses by direct contact with copper was previously demonstrated on several viruses, including *Novovirus*–the major cause of gastroenteritis [23] and *Influenza A* [23]. However, the mechanism of contact killing of viruses by copper is still not fully understood. The efficient and fast (several minutes) contact killing by copper was previously demonstrated for *monkeypox* and *vaccinia* viruses [24]. Electron microscopy studies of murine *Novovirus* placed on the surface of Copper alloys suggested that the fast virus inactivation was due to a massive breakdown of the viral capsid, which allowed the access of Cu^2+^ ions to the viral genome, resulting in a substantial reduction in the viral gene copy number [33]. As observed in our case, the efficient inactivation of viruses by contact with Cu and CuO nanoparticles does not seem to stem from Cu^2+^ ion release since no traces of copper were detected by ICP.

Besides the mechanism of virus killing, an intriguing finding is the cumulative effect of a mix of two nanoparticles–Cu and CuO–in the inactivation of viruses when compared to that of each of the nanoparticles alone for the same concentration. Very little is known so far about the antiviral mechanisms of each of the nanoparticles. In contrast, the antibacterial activity of polymer composites with these nanoparticles has been extensively studied (https://www.sciencedirect.com/science/article/pii/S0928493116308438), often suggesting a different mechanism for each. For instance, the antibacterial function of polymer composites with CuO nanoparticles was interpreted as stemming from by the large number of reactive oxygen species (ROS) that they release [34]. On the other hand, Cu nanoparticles embedded into polymer were shown to release Cu^2+^ ions that directly interact with bacteria [20]. It is possible that the two types of nanoparticles also kill viruses by different mechanisms, and that their combination produces an optimal antiviral effect, whose details are still to be studied.

In summary, we demonstrated the effective elimination of surface adsorbed viruses using surface coatings based on nanocomposite materials containing a polymer matrix and Cu/CuO nanoparticles. We showed that while each of these nanoparticle types can inactivate two types of viruses, the efficiency of the deactivation depends on the amount of the nanoparticles, with a 5% concentration being the threshold for effective virus elimination. The antiviral effect, which most probably stems from a “contact killing” mechanism, highly depends on the type of polymer, and specifically, on its affinity for the nanoparticles that determine whether the nanoparticles will remain within the polymer bulk or accumulate at the surface, where they can directly contact the adsorbed viruses. The amount and the composition of the composite can be tuned to determine the efficiency of the HCoV-OC43 inactivation. We further demonstrated the versatility of antiviral nanocomposites by showing their inactivation of a lentivirus, as well as their long-term stability through aging tests. All these open a pathway to the rational design of nanomaterial-based surface coatings aimed the minimizing contact-induced transmission of SARS-CoV-2, as well as of other viruses and bacteria.

## Materials and methods

### 1. Human coronavirus HCoV-OC43 production and stock

HCoV-OC43 virus was originally isolated in 1967 [27]and the strain employed in this study was isolated in Israel in 2008 from nasopharyngeal/oropharyngeal patient samples as described [35] under approval by the Helsinki Committee for Research on Human Beings of the Soroka Medical Center; all participants gave signed informed consent to participate and for future studies. HCoV-OC43 viral genome was extracted from 500 μL of patient samples using the NucliSENS easyMAG kit (BioMerieux, Marcy-l’Étoile, France). To determine the HCoV-OC43, all samples were subjected to qRT-PCR, as previously described [35] HCT8 cells were cultured with HCoV-OC43 in complete RPMI media containing 10% FBS, pen-strep, HEPES, L-glutamine, non-essential amino acids and sodium pyruvate (all from Biological Industries, Beit Haemek, Israel) in a humidified 5% CO_2_ 37°C incubator. Following 5 days of incubation, supernatant was collected, and cell debris were discarded by centrifugation. The supernatant was aliquoted to 0.5 ml low bind tubes and kept at -80°C. Stock was tested on HCT8 cells.

### 2. Fabrication

The preparation of the Cu/CuO polymer nanocomposites was inspired by previous work by others [12, 36]. The solutions with the various polymers were prepared by mixing copper and copper oxide nanoparticles (size: 25–50 nm, Sigma Aldrich). The concentrations were determined w/w with the amount of solids in the polymers. PMMA includes 8% solids (A-8,495K, EM Resist), and epoxy (SU-8, EM Resist) contains 50% solids. Solutions were prepared in various concentrations between 1–20% of nanoparticles. 25% of Sodium dodecyl sulfate (SDS) was added to the PMMA solutions for better mixing. All the solutions were sonicated for 1 hour. Glass substrates were cleaned by oxygen plasma for 1 min and were coated with the solutions by spin coating (speed: 3000 rpm, time: 45 sec). After spin coating, the samples were baked for 2 min at 160°C.

### 3. Aging process

The samples were aged in cycles of heating and cooling for seven days. Each cycle was 24 hours in total, 12 hours of heating at a temperature of 50°C, and 12 hours at room temperature.

### 4. Cells and tissue culture

HCT8 (ATCC no. CCL-244) and JURKAT (ATCC no. TIB-152) cells were grown in RPMI media (Gibco) containing 10% FBS, pen-strep, HEPES, L-glutamine, non-essential amino acids, and sodium pyruvate (all from Biological Industries, Beit Haemek, Israel). All tissue cultured cells were kept in a humidified 5% CO_2_ 37°C incubator.

### 5. Lentivirus (LVs) production and stock

7.5x106 HEK293T cells were plated onto poly K coated 10 cm plates (Sigma) in DMEM media containing 10% FBS, pen-strep, HEPES, L-glutamine, non-essential amino acids and sodium pyruvate (all from Biological Industries). 10 μg of pHAGE2 lenti vector and 3 μg of packaging plasmids Tat, Rev, Hgpm2 and VSV-G in a 1:1:1:2 ratio were transfected using JetPrime® reagent (Polyplus) according to the company’s protocol. Medium was replaced 4 hrs after the transfection with fresh complete DMEM. Fresh media was added on days 2 and 3 after transfection and collected on day 4. The media containing LVs was filtered through 0.45 μm PVDF membrane and aliquoted as supernatant or transferred into ultra-centrifuge tubes (Beckman Coulter) and centrifuged for 90 minutes at 17,000 RPM 4°C. The LV’s pellets were suspended, aliquoted and kept at -20°C.

### 6. Exposure of virus to the coating and subsequent transduction of target cells

#### HCoV-OC43

Two ml of viral stock, diluted with 18 μL of plain RPMI, were added to the center of cover glass (placed in well of 24-well plate) pre-coated with NP-polymer, polymer alone, or to a non-coated cover glass. Plates were dried for 2 hrs in the laminar table and then 160 μL of complete RPMI were added for 30 minutes incubation in a humidified 5% CO_2_ 37°C incubator. Then, media was harvested and added to 24-well plate containing pre-seeded HCT8 cells (150×10^3^ cells/well). After incubation for 1 h with period shaking, complete RPMI was added to the plate following additional 48 hrs incubation, RNA was extracted using magLEAD kit. Viral levels were detected using qRT-PCR.

### 7. Retrovirus (pHAGE2 GFP-lenti vector)

Twenty μL of viral stock, diluted with 10 μL of plain RPMI, were added to the center of cover glass (placed in well of 24-well plate) pre-coated with NP-polymer, polymer alone, or to a non-coated cover glass. Plates were dried for 2 hrs in the laminar table and then 500 μL of complete RPMI were added for 30 minutes incubation in a humidified 5% CO_2_ 37°C incubator. Then, half of the media was harvested and added to new 24-well plate followed by the addition of Jurkat cells (100×10^3^ cells/well). The plate was incubated for 48 hrs, cells were harvested and checked by FACS for ZsGreen expression.

## Supporting information

S1 File (DOCX)
