# Supplementary material for: Nanocomposite coatings for the prevention of surface contamination by coronavirus
Source: PLoS One. 2022 Aug 2;17(8):e0272307. doi: 10.1371/journal.pone.0272307 (PMC9345348; doi:10.1371/journal.pone.0272307)
Supplement: S1 File — (DOCX) [file pone.0272307.s001.docx]

**Supporting Information**

**Figure 1b**

| concentration | 0 | 1 | 5 | 10 | 20 |
| --- | --- | --- | --- | --- | --- |
| Fold changes | 1.361314 |  | 0.01256 | 0.011965 | 0.023931 |
|  | 0.917004 | 0.182378 | 0.014428 | 0.007164 | 0.013003 |
|  | 2.857988 | 0.410371 | 0.028067 | 0.014229 | 0.015571 |
|  | 0.82645 | 0.377618 | 0.077214 | 0.009131 | 0.018136 |

| Tukey's multiple comparisons test | Mean Diff. | 95.00% CI of diff. | Significant? | Summary | Adjusted P Value |
| --- | --- | --- | --- | --- | --- |
| 0 vs. 1 | 1.167 | 0.1241 to 2.210 | Yes | * | 0.0253 |
| 0 vs. 5 | 1.458 | 0.4919 to 2.423 | Yes | ** | 0.0026 |
| 0 vs. 10 | 1.480 | 0.5143 to 2.446 | Yes | ** | 0.0023 |
| 0 vs. 20 | 1.473 | 0.5073 to 2.439 | Yes | ** | 0.0024 |
| 1 vs. 5 | 0.2904 | -0.7528 to 1.334 | No | ns | 0.9041 |
| 1 vs. 10 | 0.3128 | -0.7303 to 1.356 | No | ns | 0.8788 |
| 1 vs. 20 | 0.3058 | -0.7373 to 1.349 | No | ns | 0.8871 |
| 5 vs. 10 | 0.02245 | -0.9433 to 0.9882 | No | ns | >0.9999 |
| 5 vs. 20 | 0.01541 | -0.9504 to 0.9812 | No | ns | >0.9999 |
| 10 vs. 20 | -0.007038 | -0.9728 to 0.9587 | No | ns | >0.9999 |

**Figure 1c**

| Concentration | 0 | 1 | 5 | 10 | 20 |
| --- | --- | --- | --- | --- | --- |
| Fold changes | 1.361314 | 1.735077 | 0.016805 | 0.012302 | 0.135372 |
|  | 0.917004 | 1.872544 | 0.342696 | 0.014033 | 0.015357 |
|  | 2.857988 | 0.803851 | 0.512278 | 0.01525 | 0.013094 |
|  | 0.82645 |  |  |  | 0.014033 |

| Tukey's multiple comparisons test | Mean Diff. | 95.00% CI of diff. | Significant? | Summary | Adjusted P Value |
| --- | --- | --- | --- | --- | --- |
| 0% vs. 1% | 0.02020 | -6.708 to 6.749 | No | ns | >0.9999 |
| 0% vs. 5% | 1.200 | -2.659 to 5.059 | No | ns | 0.3770 |
| 0% vs. 10% | 1.477 | -2.893 to 5.846 | No | ns | 0.3349 |
| 0% vs. 20% | 1.446 | -1.071 to 3.964 | No | ns | 0.1952 |
| 1% vs. 5% | 1.180 | -2.263 to 4.623 | No | ns | 0.3282 |
| 1% vs. 10% | 1.457 | -1.131 to 4.044 | No | ns | 0.1456 |
| 1% vs. 20% | 1.426 | -0.8869 to 3.739 | No | ns | 0.1237 |
| 5% vs. 10% | 0.2767 | -0.8353 to 1.389 | No | ns | 0.5011 |
| 5% vs. 20% | 0.2461 | -1.080 to 1.572 | No | ns | 0.6712 |
| 10% vs. 20% | -0.03060 | -0.3314 to 0.2702 | No | ns | 0.9161 |

**Figure 1d**

|  |  |  |  |  |  |  |  |
| --- | --- | --- | --- | --- | --- | --- | --- |
|  | PMMA | | | | Epoxy | | |
| 1% | 6.211751 | 0.182378 | 0.410371 | 0.377618 | 253.3521 | 253.3521 | 295.0877 |
| 5% | 0.01256 | 0.014428 | 0.028067 | 0.077214 | 264.1109 | 297.1402 | 243.0316 |

| Fixed effects (type III) | P value | P value summary | Statistically significant (P < 0.05)? |
| --- | --- | --- | --- |
| Row Factor | 0.9612 | ns | No |
| Column Factor | <0.0001 | **** | Yes |
| Row Factor x Column Factor | 0.9072 | ns | No |

**Figure 3d**

|  | Fresh solution | | | Aged solution | | |
| --- | --- | --- | --- | --- | --- | --- |
| CuO 5 | 0.088388 | 0.040386 | 0.097396 | 0.033262 | 1.918528 | 0.032804 |
| Cu 5 | 0.044811 | 0.189465 | 0.348686 | 0.76313 | 0.040667 | 0.033726 |
| CuO 2.5 Cu 2.5 | 0.034915 | 0.064704 | 0.02936 | 0.044502 | 0.039555 | 0.036398 |

| Source of Variation | % of total variation | P value | P value summary | Significant? |
| --- | --- | --- | --- | --- |
| Interaction | 8.366 | 0.5365 | ns | No |
| Row Factor | 8.964 | 0.5144 | ns | No |
| Column Factor | 6.164 | 0.3449 | ns | No |

**Figure 3e**

|  | Fresh coating | | | Aged coating | | |
| --- | --- | --- | --- | --- | --- | --- |
| CuO 5 | 0.088388 | 0.040386 | 0.097396 | 0.05672 | 0.071298 | 0.045753 |
| Cu 5 | 0.044811 | 0.189465 | 0.348686 | 0.044194 | 0.095391 | 0.062068 |
| CuO 2.5 Cu 2.5 | 0.034915 | 0.064704 | 0.02936 | 0.030607 | 0.033726 | 0.032577 |

| Source of Variation | % of total variation | P value | P value summary | Significant? |
| --- | --- | --- | --- | --- |
| Interaction | 12.48 | 0.2575 | ns | No |
| Row Factor | 26.56 | 0.0751 | ns | No |
| Column Factor | 11.75 | 0.1163 | ns | No |

**Figure 4a**

| concentration | 0 | 1 | 5 | 10 | 20 |
| --- | --- | --- | --- | --- | --- |
|  | 15.56 | 13.2 | 19.62 | 0.41 | 1.01 |
|  | 12.83 | 12.71 | 8.69 | 0.51 | 0.42 |
|  | 18.1 | 11.57 | 1.67 | 4.23 | 2.14 |

| Tukey's multiple comparisons test | Mean Diff. | 95.00% CI of diff. | Significant? | Summary | Adjusted P Value |
| --- | --- | --- | --- | --- | --- |
| 0 vs. 1 | 3.003 | -8.708 to 14.71 | No | ns | 0.9106 |
| 0 vs. 5 | 5.503 | -6.208 to 17.21 | No | ns | 0.5585 |
| 0 vs. 10 | 13.78 | 2.069 to 25.49 | Yes | * | 0.0203 |
| 0 vs. 20 | 14.31 | 2.595 to 26.02 | Yes | * | 0.0162 |
| 1 vs. 5 | 2.500 | -9.211 to 14.21 | No | ns | 0.9513 |
| 1 vs. 10 | 10.78 | -0.9348 to 22.49 | No | ns | 0.0751 |
| 1 vs. 20 | 11.30 | -0.4081 to 23.01 | No | ns | 0.0597 |
| 5 vs. 10 | 8.277 | -3.435 to 19.99 | No | ns | 0.2137 |
| 5 vs. 20 | 8.803 | -2.908 to 20.51 | No | ns | 0.1729 |
| 10 vs. 20 | 0.5267 | -11.18 to 12.24 | No | ns | 0.9999 |

**Figure 4b**

| concentration | 0 | 1 | 5 | 10 | 20 |
| --- | --- | --- | --- | --- | --- |
|  | 15.56 | 13.82 | 0.43 | 0.98 | 0.95 |
|  | 12.83 | 3.57 | 0.5 | 1.4 | 0.14 |
|  | 18.1 | 15.51 | 1.95 | 0.81 | 0.11 |

| Tukey's multiple comparisons test | Mean Diff. | 95.00% CI of diff. | Significant? | Summary | Adjusted P Value |
| --- | --- | --- | --- | --- | --- |
| 0 vs. 1 | 4.530 | -3.946 to 13.01 | No | ns | 0.4445 |
| 0 vs. 5 | 14.54 | 6.060 to 23.01 | Yes | ** | 0.0016 |
| 0 vs. 10 | 14.43 | 5.957 to 22.91 | Yes | ** | 0.0016 |
| 0 vs. 20 | 15.10 | 6.620 to 23.57 | Yes | ** | 0.0012 |
| 1 vs. 5 | 10.01 | 1.530 to 18.48 | Yes | * | 0.0199 |
| 1 vs. 10 | 9.903 | 1.427 to 18.38 | Yes | * | 0.0212 |
| 1 vs. 20 | 10.57 | 2.090 to 19.04 | Yes | * | 0.0143 |
| 5 vs. 10 | -0.1033 | -8.580 to 8.373 | No | ns | >0.9999 |
| 5 vs. 20 | 0.5600 | -7.916 to 9.036 | No | ns | 0.9994 |
| 10 vs. 20 | 0.6633 | -7.813 to 9.140 | No | ns | 0.9989 |
